# Multifaceted roles of centrosomes in development, health, and disease

**DOI:** 10.1093/jmcb/mjab041

**Published:** 2021-07-15

**Authors:** Feifei Qi, Jun Zhou

**Affiliations:** 1 Institute of Biomedical Sciences, College of Life Sciences, Shandong Normal University, Jinan 250014, China; 2 State Key Laboratory of Medicinal Chemical Biology, College of Life Sciences, Nankai University, Tianjin 300071, China

**Keywords:** centrosome, germ cell, stem cell, immunity, ciliopathy

## Abstract

The centrosome is a membrane-less organelle consisting of a pair of barrel-shaped centrioles and pericentriolar material and functions as the major microtubule-organizing center and signaling hub in animal cells. The past decades have witnessed the functional complexity and importance of centrosomes in various cellular processes such as cell shaping, division, and migration. In addition, centrosome abnormalities are linked to a wide range of human diseases and pathological states, such as cancer, reproductive disorder, brain disease, and ciliopathies. Herein, we discuss various functions of centrosomes in development and health, with an emphasis on their roles in germ cells, stem cells, and immune responses. We also discuss how centrosome dysfunctions are involved in diseases. A better understanding of the mechanisms regulating centrosome functions may lead the way to potential therapeutic targeting of this organelle in disease treatment.

## Introduction

The centrosome is a conserved organelle located near the center of animal cells that is ∼1‒2 μm in diameter (Stearns and Winey, 1997; Hoyer-Fender, 2012). It consists two centrioles characterized by a nine-fold radial arrangement of microtubules surrounded by pericentriolar material (PCM), an ordered structure comprising at least two layers. The two centrioles have distinct structures and functions because of their generational difference. The older mother centriole is fully matured and distinguished by distal and subdistal appendages, while the daughter centriole arises from centrosome duplication during the previous cell cycle. Only the mother centriole can turn into a basal body, which is required for cilium and flagellum formation (Joukov and De Nicolo, 2019). Some PCM proteins, such as spindle-defective protein 5 in the nematode *Caenorhabditis elegans* and centrosomin in *Drosophila*, are reported to undergo liquid‒liquid phase separation, and, therefore, the centrosome is expected to behave as a biomolecular condensate (Woodruff et al., 2017; Rale et al., 2018). However, the concept of a liquid-like centrosome is not widely accepted, because some studies suggest that centrosomes are assembled upon a more solid, stable scaffold (Raff, 2019). As an important organelle, the centrosome is involved in a variety of cellular processes.

The best-known function of centrosomes is as a microtubule-organizing center (MTOC) that organizes interphase microtubules and mitotic spindles. During interphase, microtubules are organized in astral arrays that emanate from the centrosome and serve as a scaffold for organelle and vesicle trafficking. During mitosis, the centrosome aids in, but is not essential for, the assembly of the bipolar spindle, which is important for accurate chromosome segregation (Khodjakov et al., 2000; Varmark, 2004). Centrosomes are duplicated during S phase in coordination with DNA synthesis. Thus, when cells enter the M phase, they contain two centrosomes that nucleate microtubules to form a bipolar spindle apparatus. Recently, there has been significant progress in our understanding of the roles of centrosomes beyond the MTOC. It is increasingly regarded as a communication hub for signaling molecules. For example, the centrosome is involved in the ubiquitin proteasome pathway for protein degradation. Immunofluorescence analyses have revealed the accumulation of core proteasome components, including the 19S and 20S subunits, at the centrosome, where they colocalize with γ-tubulin (Wigley et al., 1999). Additionally, the centriole-derived basal body is required for the formation of cilium, an important organelle that senses extracellular signals. Accordingly, abnormalities in centrosomes perturb signal transduction and can lead to ciliopathies (Bettencourt-Dias et al., 2011).

Given its involvement in various cellular processes, it is unsurprising that centrosome abnormalities in the number or structure result in diseases and disorders that exhibit cell type-specific characteristics. The link between aberrant centrosome number and cancer development has been known for many years, but the molecular basis is only now being elucidated (Levine et al., 2017; Raff and Basto, 2017). Upon fertilization, centrioles are inherited from sperm, while most centrosomal proteins are derived from oocytes. Thus, dysfunction of centrosome components in gametes may lead to fertility problems and abnormal embryonic development (Sha et al., 2017). Centrosome dysfunction has also been implicated in brain developmental diseases, such as Alstrom syndrome and Bardet‒Biedl syndrome (Rauch et al., 2008; Nano and Basto, 2017). Centrosome translocation in immune cells is crucial for targets killing, and it was recently demonstrated that centrosome abnormalities result in defects in immunity (Stinchcombe and Griffiths, 2014; Wu et al., 2020). In this review, we summarize the current state of knowledge regarding the roles of centrosomes and centrioles in the context of the reproductive system, stem cells, as well as immunity, and we discuss concisely diseases and disorders caused by centrosome abnormalities, including neurodevelopmental disorders and ciliopathies. Understanding completely the molecular mechanisms by which centrosome aberrations result in human diseases can provide a basis for the development of new treatments.

## Centrosomes in germ cells and centrosome abnormalities in infertility

### The centrosome of germ cells

In most non-rodent mammalian species, including humans, it is the sperm that contributes centrioles to the zygote, while the centrioles in oocytes are destroyed, a process to ensure an appropriate zygotic centriole number (Simerly et al., 1995; Palermo et al., 1997). The theory of uniparental distribution of the centrosome was first raised by Theodor Boveri in 1901 through utilizing sea urchin to show that the egg loses the centrosome during oogenesis whereas the sperm contains this structure. The dogma that the sperm donates centrioles to the embryo is not applicable to mice and other murine animals who lose centrioles completely during spermiogenesis. However, the potential explanations for the lack of centrioles in the sperm of mice is not included in our review (please see Avidor-Reiss and Fishman, 2019). Studies of centrosome structure and behavior in germ cells reveal that centrosome dynamics differ in oocytes and sperm cells.

**Figure 1:**
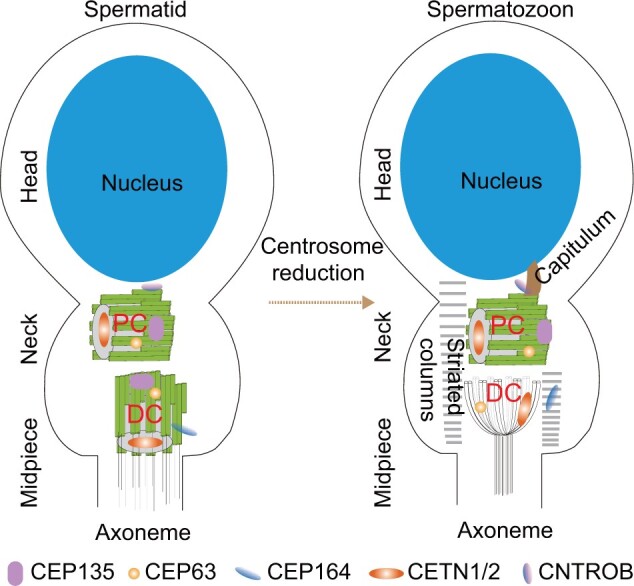
The sperm centrosome is remodeled during spermatogenesis. Initially, there are two centrioles located in the neck of spermatid cell. Both the PC, which articulates with the sperm nucleus, and the DC are composed of barrel-shaped microtubules. Later during spermatogenesis, the DC is remodeled through centrosome reduction into a structure consisting of splayed microtubules, and the PCM transforms into the capitulum and striated columns. The centriolar lumen protein CETN1/2 and the PCM protein CEP63 localize at the DC, while the centriole wall protein CEP135 and the appendage protein CEP164 are lost. The protein CEP164 instead localizes to the striated columns. The PC is slightly altered in mature spermatozoa. It maintains the typical centriole structure and the centriolar proteins CEP135 and CETN1/2, the PCM protein CEP63, and the appendage protein CEP164. The centriole wall protein CNTROB is missed from the centriole wall but localizes to the capitulum.

The mature human sperm contains two centrioles, a typical centriole and an atypical centriole, that are conveyed to the zygote (Figure 1). The typical centriole, which is also named proximal centriole (PC), is located within the connecting piece next to the basal plate of the sperm head with barrel-shaped structure of nine triplet microtubules embedded in pericentriolar components. The atypical centriole, which is referred to distal centriole (DC) as well, lies perpendicular to the PC and is composed of microtubules in a splayed arrangement (Fishman et al., 2018). The two centrioles and their surrounding PCM are remodeled through a process of centrosome reduction during spermiogenesis (Manandhar and Schatten, 2000; Avidor-Reiss et al., 2015). During DC reduction, only residual microtubules are preserved while the typical structure disintegrates and most PCM proteins are eliminated. The PCM transforms into specialized structures, the capitulum and striated columns. The DC lacks the centriolar wall protein CEP135 and the appendage protein CEP164. The protein CEP164 instead localizes to the striated columns. The DC has rods made of the centriolar lumen protein CETN1/2 and the PCM protein CEP63 (Fishman et al., 2018). A recent study revealing the role of Poc1 proteins in sperm demonstrated that the atypical DC functions as the zygote’s second centriole and is required for normal fertility and embryonic development in *Drosophila* (Khire et al., 2016). A recent paper showed that, in mammalian sperm, the atypical DC and its surrounding atypical pericentriolar matrix form a dynamic basal complex that facilitates a cascade of microtubule sliding deformations in the axoneme, coupling tail beating with asymmetric head kinking (Khanal et al., 2021). The PC alters slightly and is introduced into the oocyte, contributing to the formation of sperm aster that is essential for uniting sperm and oocyte pronuclei (Meaders and Burgess, 2020). The PC maintains the typical centriole structure and the centriolar proteins CEP135 and CETN1/2, the PCM protein CEP63, and the appendage protein CEP164. The centriolar wall protein CNTROB (Centrobin, the centriole duplication, and spindle assembly protein) is missing from the centriolar wall but localizes to the capitulum. Recently, a paper demonstrated that the centrosome, located at the interface between the two pronuclei, was associated with chromosomes and determined the site of chromosome clustering and accuracy of chromosome segregation (Cavazza et al., 2021).

The oogonia originally contain centrioles that are eliminated during oogenesis, yielding a mature oocyte lacking centrioles; however, PCM and centrosome-associated proteins, including pericentrin (PCNT), γ-tubulin, and nuclear mitotic apparatus, are retained (Hoyer-Fender, 2012). Centriole elimination is essential for avoiding parthenogenesis, although the precise molecular mechanisms involved are largely unknown. Centrioles are maintained up to the pachytene stage of meiosis in *C. elegans*, mouse, rat, and human and are absent in subsequent stages (Mikeladze-Dvali et al., 2012). In *Drosophila*, oocytes inherit the contents of supportive nurse cells, including their centrioles, which form an aggregate known as the ‘centriolar complex’ (Mahowald and Strassheim, 1970). The centriolar complex is reported to be eliminated in late oogenesis, a process triggered by reduction in Polo-like kinase (Plk) activity (Pimenta-Marques et al., 2016). In starfish oocytes, three centrioles are selectively removed by extrusion into polar bodies, while the sole remaining daughter centriole is degraded in anaphase II, and this process is independent of Plk1 (Borrego-Pinto et al., 2016; Pierron et al., 2020). To sum up, although centriole elimination is a hallmark feature of oocytes, the mechanism manipulating the process varies. Undoubtedly, disturbed centriole elimination may cause supernumerary centrioles in the embryo, and thus resulting in unsuccessful embryonic development.

### Centrosome aberrations in infertility

Because of the centriole’s essential functions, such as forming the sperm tail during spermiogenesis, linking the sperm head and tail, mediating pronuclear migration, it is expected that defects in sperm centrioles can cause a spectrum of diseases, e.g. oligozoospermia, asthenozoospermia, teratozoospermia, and acephalic spermatozoa syndrome (Avidor-Reiss et al., 2020). One of the roles of the sperm centrioles is to push the sperm head to the oocyte center and promote the fusion of male and female pronuclei, via nucleating and organizing the sperm aster. Therefore, centrosomal defects will cause fertilization failure and developmental arrest at the pronuclear stage (Rawe et al., 2002; Chatzimeletiou et al., 2008).

The role of the centrosome in linking the head of sperm, which contains the genetic material, and the tail, which generates the force for swimming, indicates that this organelle is important for healthy sperm. Studies have consistently shown that morphological defects/physically separated sperm segments (head only, head and tail separated, or isolated tail) compromise centrosome functions in the zygotes (Schatten and Sun, 2009). There is accumulating evidence that centriole abnormalities result in morphological and molecular defects that contribute to sperm-derived infertility (Schatten and Sun, 2009). Several mutations in centriolar protein-encoding genes have been identified in mammals that result in the failure of this head‒tail connection, such as *Centrin 1* (*CETN1*), *CNTROB*, and *TSGA10* (Liska et al., 2009; Avasthi et al., 2013; Sha et al., 2018). CETN1 is specifically enriched in sperm cells. Germline deletion of *Cetn1* in mice induces spermatids to lack tails (Avasthi et al., 2013; Moretti et al., 2017). CNTROB is a daughter centriole-specific protein. Mutant spermatids that express truncated CNTROB protein exhibit defective head‒tail linkage (Liska et al., 2009). *TSGA10* is expressed solely in testis. Mouse *Tsga10* encodes a 65-kDa spermatid protein that appears to be processed to a 27-kDa protein within the fibrous sheath, a major sperm tail structure. A patient with a homozygous deletion within TSGA10 (A71Hfs*12) showed 99% headless sperm in the ejaculate (Modarressi et al., 2004; Sha et al., 2018). In *Drosophila*, appropriate proximal end docking to the nucleus is dependent on the restriction of PCNT-like protein (PLP) and PCM to the proximal end of both centrioles (Galletta et al., 2020). Ectopic positioning of PLP to more distal portions of the centriole results in erroneous, lateral centriole docking to the nucleus, and this causes sperm decapitation as a result of defective head‒tail linkage.

Another role of the centriole in sperm is to form the flagellum of the sperm tail. In *Drosophila*, multiple mutations in centriole genes have shown defects in sperm flagellum formation (Khire et al., 2016; Reina et al., 2018). Recently, two centriolar proteins, CEP135 and DZIP1, have been identified with multiple morphological abnormalities of the sperm flagella (MMAF) in infertile males (Sha et al., 2017; Lv et al., 2020). CEP135 is a centriole core protein located in the cartwheel and centriole wall (Kraatz et al., 2016). A homozygous missense mutation (p. D455V) in CEP135 was shown to result in severe MMAF where only 60% of the sperm had flagellum and 45% were short (Sha et al., 2017). DZIP1 is a component of the distal appendage, which functions in microtubule anchoring as well as anchoring the centriole to the cell membrane during cilium formation (Zhang et al., 2017; Lapart et al., 2019). A homozygous missense mutation (p. R63Q) or a homozygous truncation mutation (p. Y230*) in DZIP1 was shown to induce asthenoteratospermia with severe MMAF (Lv et al., 2020). To understand centrosome-induced fertilization defects, it is important to understand the centriole‒centrosome complex and its regulation and function during fertilization and embryonic development. A comprehensive understanding of the centriole is also essential for an effective diagnosis. Heterologous intracytoplasmic sperm injection can be used to correct specific sperm-related centrosome dysfunctions at a molecular level (Schatten and Sun, 2009).

Although the importance of centrosomes in sperm cells is widely acknowledged, there are many open questions, including the composition and regulatory mechanisms of centrosomes in sperm and fertilized and unfertilized oocytes. Moreover, identifying centrosome-associated proteins at each stage of fertilization could aid in screening sperm cells for improved success rates with *in vitro* fertilization.

## Centrosomes and asymmetric cell division in stem cells

Asymmetric cell division (ACD) is a fundamental process employed by many stem cells to maintain tissue homeostasis by producing one stem cell and one differentiated cell (Morrison and Kimble, 2006; Inaba and Yamashita, 2012). During this process, many cellular components, including cell fate determinants, damaged proteins, and some organelles, are asymmetrically inherited (Chen et al., 2016a). The inherent asymmetry of centrosomes is thought to play a key role in establishing cellular asymmetry and in determining the mitotic axis of stem cells undergoing ACD.

**Figure 2:**
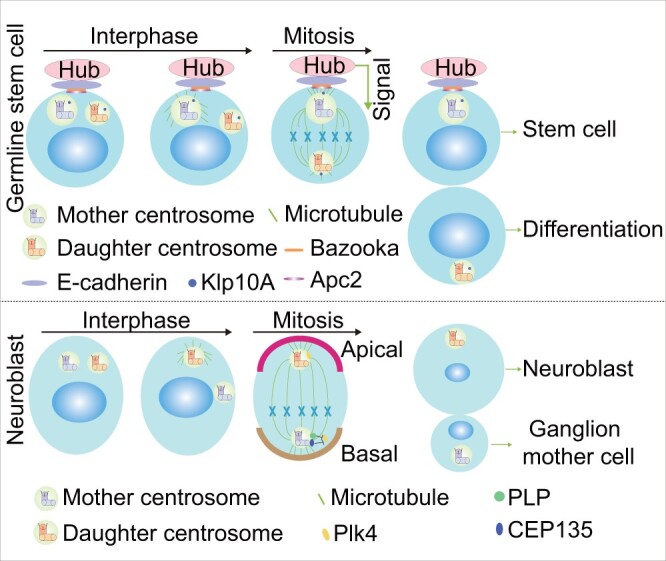
Centrosomes and asymmetric stem cell division. During asymmetric division of GSCs, the mother centrosome is connected to the hub via adherens junctions in conjunction with Apc2, Bazooka, and Klp10A. In response to signals from the hub, GSC inherits the mother centrosome and retains a stem cell identity, while the sibling cell inherits the daughter centrosome and undergoes differentiation. In contrast, during asymmetric division in neural stem cells, the neuroblast inherits the daughter centrosome, while the ganglion mother cell inherits the mother centrosome. This may involve inhibition of mother centrosome activity by PLP and CEP135, which block Plk4 recruitment.

Up to present, most of our knowledge of stem cell centrosomes comes from studies in *Drosophila* and *C. elegans*, given the difficulty in using mammalian systems to study the functions of centrosomes in stem cell division and differentiation (Gallaud et al., 2017; Pacquelet, 2017). For example, mother and daughter centrosomes containing the older and younger mother centrioles, respectively, were determined to have distinct fates, using male *Drosophila* as a model system (Yamashita and Fuller, 2008). Germline stem cells (GSCs) preferentially retain the mother centrosome to maintain stem cell characteristics, while sibling cells inherit the daughter centrosome to differentiation (Figure 2). Although the detailed mechanisms of fate-specific centrosome segregation are unclear, differences in microtubule nucleation capacity between mother and daughter centrosomes and associated upstream proteins are thought to be important (Venkei and Yamashita, 2018). The older centrosome accumulates more PCM than the younger one and then is anchored next to the hub, from which it receives signals that preserve its stem cell identity. Furthermore, membrane-localized adenomatous polyposis coli 2 (Apc2) and Bazooka were reported to make contributions to this process by interacting with E-cadherin and tethering the mother centrosome next to the hub, thus ensuring asymmetric stem cell division (Yamashita et al., 2003; Inaba et al., 2015). Additionally, centrosome-localized kinesin-like protein at 10A (Klp10A), a microtubule-depolymerizing kinesin of the kinesin-13 family, was shown to regulate centrosomes in GSCs. *Klp10A* depletion yielded a larger GSC and a smaller differentiating cell as a result of mother centrosome elongation, demonstrating that centrosome behavior must be strictly controlled during ACD (Chen et al., 2016b).

Asymmetric centrosome distribution has also been observed in *Drosophila* larval neural stem cells known as neuroblasts, which generates a larger self-renewing neuroblast and a smaller ganglion mother cell that undergoes differentiation. Unlike in male germ cells, it is the daughter centrosome that attaches to the neuroblast cortex, equipped with the abilities to maintain centrosome material and organize microtubules, while the mother centrosome moves away and is inherited by the differentiating daughter cell (Figure 2; Rebollo et al., 2007; Conduit and Raff, 2010; Januschke et al., 2011, 2013). Mechanistically, the activity of the mother centrosome is inhibited by PLP and CEP135, which negatively regulate centrosome maturation and activity, by blocking the recruitment of Polo kinase (Lerit and Rusan, 2013; Singh et al., 2014). In addition, Plk4 was showed to play roles in stereotypical asymmetric centrosome dynamics through phosphorylating Spd2, which triggers the displacement of Spd2 and promotes disassembly of PCM around the mother centrosome (Gambarotto et al., 2019).

Although essential centrosome functions have been revealed by studies in *Drosophila* GSCs, the factors and mechanisms regulating stem cell centrosomes and their influence on stem cell fate remain to be determined. Additionally, studies on centrosome behavior in mammalian stem cells are still needed to determine whether the mechanisms observed in *C. elegans* and *Drosophila* are conserved in all animals. Biochemical and structural analyses may help to answer these questions.

## Centrosomes and immunity

### Centrosome translocation in immune cells

The mammalian immune system, including innate immunity and adaptive immunity, has various cell types that protect the body from infection (Tomar and De, 2014). Cytotoxic T lymphocytes (CTLs), natural killer (NK) cells, and invariant natural killer T (iNKT) cells are cytolytic immune cells that release secretory lysosomes to kill infected cells. The general mechanism of target cell killing involves secretion of lytic granules containing the hydrophobic protein perforin and several granzyme proteases (Kabanova et al., 2018; McComb et al., 2019). Perforin forms oligomeric pores on the target cell surface that allow granzymes to access the cytoplasm, where they cleave specific substrates to induce cell apoptosis. Additionally, cytokines released by cluster of differentiation 4-positive (CD4^+^) regulatory T cells activate other immune cells. The precise targeting of both secretory lysosomes and cytokines to infected cells, but not normal cells, is dependent on centrosomes (Stinchcombe et al., 2011).

**Figure 3:**
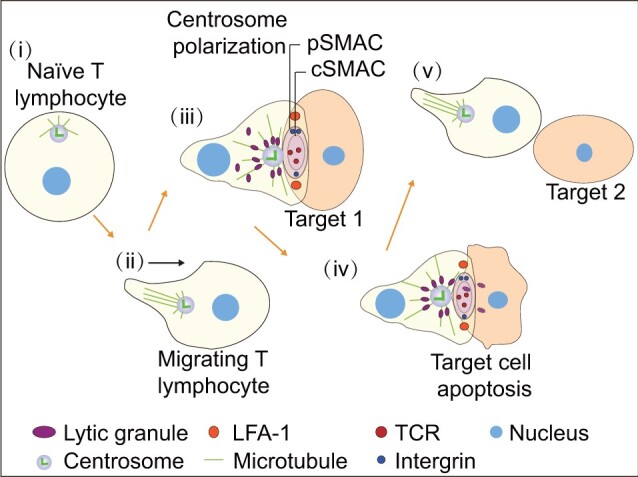
Centrosome functions in the immune response. (i) In naïve T lymphocytes, the centrosome is close to the nucleus and organizes microtubules toward the membrane. (ii) In migrating T lymphocytes, the centrosome is located at the back of nucleus. (iii) When T lymphocytes encounter a target cell, an IS, comprising a cSMAC and a pSMAC, is formed at the interface. The cSMAC contains accumulated TCRs. The activated TCR, along with lymphocyte function-associated antigen 1, triggers centrosome polarization to the IS. Microtubules emanating from the centrosome contribute to lytic granule delivery and target cell killing. (iv and v) Once the target is destroyed, the centrosome moves back into the cell body or polarizes to the next target.

When T lymphocytes interact with antigen-presenting cells, an immunological synapse (IS) is formed at the interface that comprises a central supramolecular activation complex (cSMAC) and a peripheral SMAC (pSMAC). The cSMAC contains accumulated T-cell receptors (TCRs), while the pSMAC is composed of a ring of integrins. IS formation is critical for TCR activation and is linked to centrosome repositioning, which is induced by signals from TCR and lymphocyte function-associated antigen 1 as well as increased calcium concentration (Roig-Martinez et al., 2019). The centrosome is exquisitely sensitive and able to polarize in response to very low avidity signals via the TCR (Jenkins et al., 2009). TCR signal leads to centrosome repositioning from the uropod, slightly distant from the nucleus to polarize toward the synapse. Live cell imaging experiments have demonstrated that the centrosome can oscillate back and forth at the membrane and retract, moving back into the cell body or polarizing toward the next target (Figure 3; Kuhn and Poenie, 2002; Mastrogiovanni et al., 2020). When the infected cell is destroyed, the centrosome either moves to a second target, returns to the uropod in migrating cells, or localizes near the nucleus in static cells. Timely repositioning of the centrosome from the cell surface to the back of the cell is necessary to terminate the immunological response at the time of, or prior to, cell separation (Stinchcombe and Griffiths, 2014).

The importance of centrosome movement during immune cell activation was recognized based on the observation that the centrosome moves directly to the site of contact and associates with the membrane until the infected cell is killed (Geiger et al., 1982). The exact site of centrosome polarization was found to be between the region of TCR clustering and the sites of secretory granule docking and secretion, suggesting a role in granule delivery to the secretory cleft along microtubules in the minus-end direction (Stinchcombe et al., 2006, 2011). In support of this notion, abnormalities in centrosome polarization were shown to prevent lysosome secretion. The significance of centrosome polarization was confirmed by studies in B lymphocytes in which the centrosome was ablated (Yuseff et al., 2011, 2013). B lymphocytes also form ISs and secrete lysosomes in the presence of surface-tethered antigens, and proteases released from the lysosomes promote antigen uptake. In the absence of centrosomes, microtubules are not reorganized and lysosomes are not released and delivered to target cells. NK and iNKT cells use similar mechanisms to target secretory lysosomes to the IS. Importantly, a recent study of centriole-deficient CTLs challenged the widely held view that centrosomes mediate the specific delivery of lytic granules to the IS (Tamzalit et al., 2020). In these centriole-deficient CTLs, polarized secretion of the granules was preserved, but the killing efficiency was reduced, owing to defects in both lytic granule biogenesis and synaptic actin remodeling. These data highlight an unexpected role for centrosomes in modulating the capacity, but not the specificity of cell killing. The role of centrosome translocation in immunity needs further investigation.

In addition to secretory lysosomes, centrosome repositioning at the membrane induces the accumulation of the Golgi apparatus and endocytic recycling compartments that are responsible for protein synthesis and downregulation of membrane proteins, respectively, at the IS (Bonello et al., 2004; Das et al., 2004; Soares et al., 2013). Thus, centrosome polarization can regulate communication at the synapse and promote an efficient and effective immune response. Some cytokines secreted by CD4^+^ T cells, which aid in target cell killing, such as interleukin 6 (IL-6), IL-10, and monocyte chemoattractant protein 1 (MCP1), rely on centrosome polarization (Huse et al., 2006). Disrupting centrosomes by chemically or genetically blocking centriole assembly attenuates the production of these cytokines (Vertii et al., 2016). On the other hand, some cytokines such as IL-2 and interferon-γ are released in the absence of centrosome polarization (Chemin et al., 2012). Taken together, these findings reveal the important role of centrosomes in the immune response.

### Mechanisms controlling centrosome positioning in immune cells

The mechanisms regulating centrosome movement in immune cells are gradually emerging. The TCR-activated tyrosine kinase Lck and related tyrosine kinase Fyn were shown to control centrosome translocation (Tsun et al., 2011). In cells deficient in both genes, centrosomes lose the ability to translocate and remain on the distal side of the nucleus. Consequently, lytic granules fail to release their contents, and target cells are not killed. Factors that regulate the polymerization/depolymerization of microtubules may also drive centrosome polarization. The centrosomal protein casein kinase I delta (CKIδ) was shown to control centrosome translocation to the IS, through the binding and phosphorylation of the microtubule plus-end-binding protein end-binding 1 (EB1) (Zyss et al., 2011). The CKIδ‒EB1 complex was proposed to accelerate microtubule growth speeds and generate long-stable microtubules necessary for centrosome translocation. It was reported that T cells reposition their centrosomes via a microtubule end-on capture‒shrinkage mechanism that operates at the center instead of the periphery of the IS (Yi et al., 2013). Consistent with such a mechanism, dynein attaches to the plus end of microtubules and exerts as pulling force on the centrosome through microtubule depolymerization (Laan et al., 2012). Inhibiting microtubule depolymerization or dynein blocks centrosome repositioning (Yi et al., 2013). Additionally, actin clearance from the center of synapse contributes to dynein accumulation at the IS, a process requiring diacylglycerol and protein kinase C (Sanchez et al., 2019). Besides, tripartite motif (TRIM) proteins modulate the innate immune response by regulating centrosome integrity. TRIM 43, an E3 ubiquitin ligase, suppresses the reactivation of herpesvirus by targeting centrosomal protein PCNT for degradation, which subsequently leads to the loss of nuclear envelope integrity and alterations in viral chromatin, suggesting PCNT as a potential therapeutic target in the treatment of herpesvirus infection (Full et al., 2019).

In summary, centrosomes in immune cells contribute to the immune response through polarized delivery of lytic granules and cytokines, a process that is accompanied by centrosome translocation. Less is known about the detailed mechanisms controlling centrosome behavior though actin and microtubule dynamics, which generate forces that pull the centrosome toward the IS. Furthermore, the signals that drive centrosome movement and thus initiate or terminate the immune response also warrant further exploration.

## Centrosome dysfunction in human diseases

### Centrosome dysfunction in neurodevelopmental disorders

Brain development is sensitive to aberrations in centrosome number and structure (Chavali et al., 2014). To date, three autosomal recessive developmental disorders, including microcephaly primary hereditary (MCPH), seckel syndrome (SCKL), and microcephalic osteodysplastic primordial dwarfism type II (MOPDII), have been attributed to centrosome dysfunction (Woods et al., 2005; Bober and Jackson, 2017). Newborns affected by these diseases manifest reduced cerebral cortex size and intellectual disability, although the overall organization of the brain is usually unaffected (Nigg and Holland, 2018). Mutations in genes encoding proteins that regulate centrosome assembly (*CEP135*, *CEP152*, *CEP63*, *CPAP*, *STIL*, *CDK5RAP2*, *SAS6*, and *PLK4*) and maturation (*CEP152* and *CENPJ*) have been identified in MCPH patients (Bond et al., 2005; Guernsey et al., 2010; Kalay et al., 2011; Pagnamenta et al., 2012; Yigit et al., 2015). Moreover, many MCPH-related proteins, such as abnormal spindle-like microcephaly-associated protein (ASPM), have been involved in spindle positioning, which plays an important role in the development of disease (Fish et al., 2006; Johnson et al., 2018). SCKL is a type of microcephalic primordial dwarfism characterized by intrauterine growth restriction, short stature, a small head, and distinct, dysmorphic (bird-like) facial features. Some centrosome-associated genes (*CENPJ*, *CEP63*, *CEP152*, and *NIN*) are involved in SCKL (Zheng et al., 2016; Nigg and Holland, 2018). In addition, mutations in *PCNT* were dissected as a cause of SCKL (Griffith et al., 2007) and MOPD II (Rauch et al., 2008). The centrosome protein ANKA, which localizes at the subdistal appendages of the mother centriole in specific subtypes of neural stem cells and in almost all basal progenitors, regulates neurogenesis via microtubule organization (Camargo Ortega et al., 2019). However, the association between mutations in genes encoding centrosomal proteins and clinical manifestations is unclear. One possibility is that centrosome defects may impair the ACD of neuronal progenitors.

The complexity of the human brain makes it difficult to study many brain disorders in model organisms. Mouse mutants for several of the known genes, such as *Aspm* and *Cdk5rap2*, have failed to reproduce the severely reduced brain size seen in human patients (Lizarraga et al., 2010; Pulvers et al., 2010). As a result, it has been challenging to study neurodevelopmental disorders in model systems. Thanks to the recent emergence of powerful 3D *in vitro* cerebral organoid system, the human brain development and microcephaly have been successfully recapitulated *in vitro* (Lancaster et al., 2013; Setia and Muotri, 2019). Even though brain organoids present the potential to study the mechanisms of microcephaly, a recent study on the comparative transcriptomes between primary human cortical cells of unknown genetic background, disease status, and brain organ indicated that brain organoids do not entirely mimic the physiological functionality of the human brain (Pulvers et al., 2010). If we can generate a repertoire of induced pluripotent stem cells (iPSCs) from microcephaly patients, we are able to generate patient-specific 3D tissue that contributes to dissecting the mechanisms of microcephaly. In addition, genome editing to acquire disease-relevant patient mutations in pluripotent cells is an attractive alternative to patient-specific iPSCs (Gabriel et al., 2020). In summary, the emergence of 3D human brain organoids and various genomic tool kits will help us to dissect the mechanisms of microcephaly and eventually enable us to reconstruct the complex processes involved in the human brain development.

### Centrosome dysfunction in ciliopathies

The mother centriole is able to transform into a basal body that is essential for cilium formation, including primary cilium and motile cilium (Kumar and Reiter, 2020). The primary cilium, composed of nine doublets of microtubules without a central microtubule pair (‘9+0’), functions as a signaling center, while the motile cilium comprises nine doublets of microtubules with a central microtubule pair (‘9+2’) (Sun et al., 2019). One centrosome contains a pair of centrioles, termed the mother and daughter centrioles that are distinguished by the distal and subdistal appendages present on the mother centriole. When cells exit from the cell cycle and enter the quiescent G0 phase, the mature mother centriole migrates to the cell surface and docks to the plasma membrane with the help of the distal appendage and subsequently becomes a basal body that nucleates the primary cilium (Tanos et al., 2013; Stinchcombe et al., 2015). In multi-ciliated cells (MCCs), multiple motile cilia are produced dependent on the basal bodies converted from large numbers of centrioles. Most centrioles amplified by MCCs grow on the surface of organelles called deuterosomes that are composed of several proteins required for centriole duplication and can be nucleated by an existing centriole or form spontaneously in the cytoplasm (Zhao et al., 2013, 2020).

**Figure 4:**
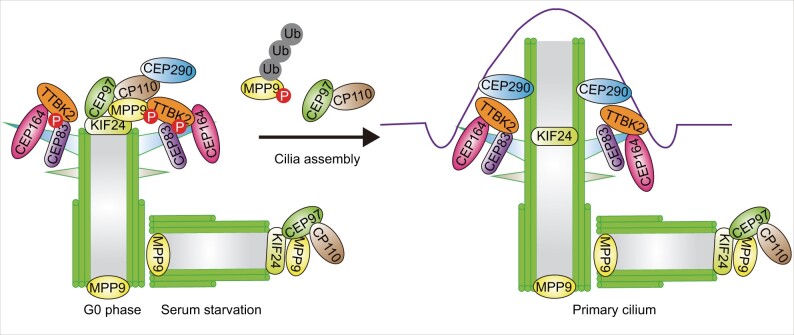
Regulation of centriole conversion to the basal body. In growing cells, CP110‒CEP97 complexes are recruited by KIF24 and MPP9 proteins to the distal ends of both centrioles, thereby inhibiting cilia assembly. In addition, CP110 antagonizes the function of CEP290, a positive regulator of ciliogenesis. When cells exit from the cell cycle and enter the G0 phase, TTBK2 is recruited by CEP164 to the mother centriole and phosphorylates MPP9 and CEP83 to promote the removal of CP110‒CEP97 complex from the mother centriole, thus initiating ciliogenesis.

Although the detailed mechanisms underlying the conversion of the mother centriole to basal body have not been fully elucidated, recent studies have shed light on the regulators that control this process (Figure 4). The centriolar coiled coil protein 110 (CP110, also known as Ccp 110), a protein that plays an essential role in centrosome duplication and cytokinesis, was identified to suppress ciliogenesis (Spektor et al., 2007). CP110 is recruited by CEP97 to the distal ends of both centrioles in non-ciliated cells. At the beginning of ciliogenesis, CP110 is removed from the mother centriole and degraded through ubiquitylation, but remains at the distal end of the daughter centriole. Loss of CEP97 or CP110 promotes primary cilium formation, suggesting that CEP97 and CP110 collaborate to inhibit ciliogenesis. In addition to CEP97, the kinesin family member 24 (KIF24), a centriolar kinesin, is another protein associated with CP110 and recruits CP110 to localize at the mother centriole (Kobayashi et al., 2011). Meanwhile, KIF24 remodels microtubules at the distal end of the mother centriole, thereby regulating cilia assembly. Loss of KIF24 results in the disappearance of CP110 from mother centrioles and accelerated primary cilia assembly in growing cells. The CP110‒CEP97 pathway has been elaborated further by recent studies showing that M-phase phosphoprotein 9 (MPP9) regulates the localization of CP110‒CEP97 to the mother centriole (Huang et al., 2018). MPP9 is recruited by KIF24 to the distal end of the mother centriole where it forms a ring-like structure and recruits CP110‒CEP97 by directly binding CEP97. Upon the initiation of ciliogenesis, MPP9 is phosphorylated by tau tubulin kinase 2 (TTBK2), whose centrosomal localization depends on the distal appendage protein CEP164. It is then degraded via the ubiquitin‒proteasome system, which facilitates the removal of CP110 and CEP97 from the distal end of the mother centriole (Cajanek and Nigg, 2014; Oda et al., 2014). CEP83 is another substrate of TTBK2 (Lo et al., 2019). The phosphorylation of CEP83 by TTBK2 is important for ciliary vesicle docking and CP110 removal. In addition, CP110 suppresses cilium formation by interacting with CEP290, a positive regulator of ciliogenesis, and antagonizing its function (Tsang et al., 2008). Interestingly, CP110 is shown to promote cilium formation *in vivo*, contrary to findings in cultured cells (Yadav et al., 2016). Depletion of CP110 results in mislocalization of core components of subdistal appendages, thereby inhibiting the fusion of recycling endosomes to basal bodies, an early step in ciliogenesis. Taken together, CP110 is involved in a complex protein network and plays a dual role during ciliogenesis. The collective evidence indicates that the mother centriole-associated positive and negative regulators are essential for cilium formation (Hergovich et al., 2009; Joo et al., 2013). However, the detailed protein molecules involved in the transforming from centriole to basal body and the mechanisms governing this process need to be further explored.

The primary cilia have been reported to regulate a number of signaling pathways, including Hedgehog (HH), G-protein-coupled receptors, canonical and non-canonical Wnt pathways, receptor tyrosine kinases such as platelet-derived growth factor 1 (PDGFRα) and insulin-like growth factor 1, and transforming growth factor-β/bone morphogenetic protein receptors (Schou et al., 2015; Anvarian et al., 2019; Nishimura et al., 2019). Due to the various roles of cilia in cell function, the absence or dysfunction of cilia results in a spectrum of disorders, namely ciliopathies. Multiple studies have shown that alterations in the basal body structure or function can result in human disorders, including reversal or randomization in body symmetry, retinal degeneration, and cystic kidney and liver diseases (Pazour et al., 2020; Ran et al., 2020). Disruption of the role of basal body in coordinating cargo trafficking results in Bardet‒Biedl syndrome, a pleiotropic disorder characterized by retinal degeneration, obesity, learning difficulties, and polycystic kidneys (Ansley et al., 2003; Kulaga et al., 2004). Defects in motile cilia always cause primary ciliary dyskinesia, such as Kartagener syndrome (Robinson et al., 2020). Other syndromes, including Alstrom syndrome, Joubert syndrome, Oral‒Facial‒Digital syndrome, and Meckel syndrome, are also induced by defective cilia (Anvarian et al., 2019). Since centrioles form basal bodies of cilia, deficiencies in centriole/basal body-associated proteins have been implicated in ciliopathies, e.g. mutations in *CEP290*, which encodes a centrosomal protein, cause pleiotropic forms of Joubert syndrome (Bettencourt-Dias et al., 2011). Due to the tight connection between centrosomes and cilia, it is not easy to distinguish which ciliopathies result from dysfunctional centrosome signaling or ciliary signaling. Thanks to the developments in the areas of genomics and proteomics, several new technologies have emerged as powerful tools to study cilia and centrioles. For example, the development of high-throughput screening using CRISPR-based gene disruption has made it possible to conduct genome-wide screens with unprecedented precision and sensitivity. The advances in electron microscopy also contribute to providing new insights into the biology of cilia and flagella. Knowledge of the disease mechanisms will open new avenues for therapeutic strategies.

## Conclusions and future perspectives

This review highlights recent progress in our understanding of the roles of centrosomes in germ cells, stem cells, and immune responses and briefly summarizes their known functions in brain development and cilium formation. Taken together, the centrosome has an evolutionarily conserved structure and protein composition in stem cells, immune cells, and ciliated cells, but not in sperm cells. The centrosome components (PC, DC, and PCM) are remodeled during spermatid differentiation. The PC of sperm cells has a normal structure but distinct protein composition, whereas the DC is distinct in both as a part of centrosome reduction process. Also, the PCM of sperm cells is replaced by the striated columns and capitulum. The existing evidence indicates that centrosomes have cell type-specific roles and that centrosome dysfunction can result in a variety of diseases and disorders. In sperm cells, the centrosome localizes to the nuclear envelope that is essential for the sperm head‒tail connection. During oogenesis, the centrioles are eliminated to avoid parthenogenesis, though the mechanisms underlying this phenomenon are mysterious. The abnormalities of centrosomes in sperm or oocytes will result in infertility. The importance of centrosomes in stem cells is emphasized by the investigations on ACD. Knowledge from *Drosophila* and *C. elegans* systems suggests that centrosomes are pivotal to balance the generation of stem cell and differentiated cell, and thus, once this balance is broken, it will result in diseases, such as cancer. Besides, recent studies have shown that the translocation of centrosomes in ISs contributes to efficient immune response. The movement of centrosomes in immune cells is tightly regulated to initiate and terminate immune responses timely. The different regulatory proteins are responsible for the distinct functions of centrosomes. For example, in ciliated cells, the removal of CEP97 and CP110 from mother centrioles is essential for ciliogenesis. The mother centrosome-localized proteins, PLP and CEP135, negatively regulate mother centrosome maturation and activity, facilitating ACD of neural stem cells. These studies indicate that the functions of centrosomes in different cell types are regulated by specific mechanisms.

Despite advances made in our understanding of centrosome functions, there are many outstanding questions. For example, it is unclear how and why some centrosomal proteins are eliminated, reduced, or enriched during centrosome remodeling in mammalian sperm. The function of centrosomes in oocytes before their elimination is also unclear. ACD of stem cells relies on centrosome asymmetry, giving rise to the questions of whether centrosomes of symmetrically dividing cells maintain asymmetry and how symmetric cell division then proceeds. Finally, little is known about the mechanisms governing centrosome dynamics in immune cells. Given the cell-type specificity of centrosome functions, different upstream signaling pathways are likely to be involved and need to be explored separately. Clarifying centrosome behavior can reveal new therapeutic targets for the treatment of diseases and disorders caused by centrosome dysfunction.

## Funding

This work was supported by the National Natural Science Foundation of China (31730050 and 32000481).

##  

**Conflict of interest:** none declared.

##  

**Author contributions:** F.Q. wrote the manuscript and drew figures. J.Z. revised the manuscript. Both authors read and approved the final version of the manuscript.
